# An Improved OMP Algorithm for Enhancing the Anti-Interference Performance of Array Antennas

**DOI:** 10.3390/s24072291

**Published:** 2024-04-04

**Authors:** Mingyuan Gao, Yan Zhang, Yueyun Yu, Danju Lv, Rui Xi, Wei Li, Lianglian Gu, Ziqian Wang

**Affiliations:** 1 College of Big Data and Intelligent Engineering, Southwest Forestry University, Kunming 650224, China; tuayuan@swfu.edu.cn (M.G.); yuyueyun103@swfu.edu.cn (Y.Y.); xirui@swfu.edu.cn (R.X.); liwei8152@swfu.edu.cn (W.L.); gulianglian@swfu.edu.cn (L.G.); wangziqian@swfu.edu.cn (Z.W.); 2 College of Mathematics and Physics, Southwest Forestry University, Kunming 650224, China; zhangyan@swfu.edu.cn

**Keywords:** masking, noise reduction, orthogonal matching pursuit (OMP), independent component analysis (ICA), array antenna, signal reconstruction

## Abstract

The demand for precise positioning in noisy environments has propelled the development of research on array antenna radar systems. Although the orthogonal matching pursuit (OMP) algorithm demonstrates superior performance in signal reconstruction, its application efficacy in noisy settings faces challenges. Consequently, this paper introduces an innovative OMP algorithm, DTM_OMP_ICA (a dual-threshold mask OMP algorithm based on independent component analysis), which optimizes the OMP signal reconstruction framework by utilizing two different observation bases in conjunction with independent component analysis (ICA). By implementing a mean mask strategy, it effectively denoises signals received by array antennas in noisy environments. Simulation results reveal that compared to traditional OMP algorithms, the DTM_OMP_ICA algorithm shows significant advantages in noise suppression capability and algorithm stability. Under optimal conditions, this algorithm achieves a noise suppression rate of up to 96.8%, with its stability also reaching as high as 99%. Furthermore, DTM_OMP_ICA surpasses traditional denoising algorithms in practical denoising applications, proving its effectiveness in reconstructing array antenna signals in noisy settings. This presents an efficient method for accurately reconstructing array antenna signals against a noisy backdrop.

## 1. Introduction

The frequency-modulated continuous-wave (FMCW) radar positioning system based on an antenna array achieves target localization by transmitting and receiving echo signals with varying frequencies due to object reflections. The determination of frequency components in the signals is a critical aspect of this technology [1,2,3]. However, in practical scenarios, the received echo signals by the antenna array are often accompanied by noise interference, making it challenging to accurately analyze the frequency and phase from the mixer [4,5]. This ultimately affects the localization accuracy of the FMCW radar positioning system [6,7,8].

Therefore, effectively reducing the interference of noise in the echo signals to achieve accurate frequency and phase have become hot research topics. Traditional methods are intermediate frequency signal filtering [9] or windowing [10] to address this issue. The discrete-time signal processing theory proposed by Oppenheim, Schafer, and Buck laid the foundation for signal sampling and reconstruction [11]. Parks and Burrus introduced methods for designing digital filters [12] to cope with the complexity of signal processing, but the complexity of filter design also increased accordingly. Hayes’s statistical digital signal processing approach emphasizes signal analysis in the presence of random interference [13] and uses probability models to describe the relationship between signals and noise. While this method can effectively reduce the interference of noise to some extent, its accuracy is not ideal. In order to achieve effective denoising of the echo signals received by the antenna array in a complex noise interference environment, more advanced techniques are needed compared to traditional methods.

Compressed sensing (CS) [14,15,16,17,18] exploits the sparsity of signals or their sparsity in a certain transform domain, breaking the limitations of the traditional Nyquist sampling theorem. By compressing signal sampling, CS allows for perfect reconstruction of signals even at sampling frequencies far below twice the highest Nyquist frequency. In the context of FMCW radar positioning systems based on antenna arrays, the sparsity of the target object relative to the positioning background [19] made compressed sensing suitable. The frequency count of the intermediate signals, formed by the echoes received by the antenna array after reflecting off the target object, is only related to the number of target objects. This aligns with the sparsity of the frequency of intermediate signals in FMCW radar systems based on antenna arrays, which is equivalent to the sparsity of the target relative to its background. This similarity makes the application of compressed sensing technology essential for signal processing in antenna array FMCW radar systems [20,21]. The literature [22] introduces a radar signal processing method based on compressed sensing, providing new approaches and technical support for reconstructing radar signals in complex environments. However, it is found that this method yields unstable results in reconstructing noisy radar signals.

The OMP algorithm [23], as a classic method in compressed sensing, has been widely studied due to its advantages, such as low algorithm complexity, fast computation speed, and high reconstruction accuracy for sparse signals. However, it is sensitive to noise, particularly in scenarios where the reconstructed signal contains substantial noise interference, leading to unstable reconstruction results. Additionally, the algorithm’s performance in reconstructing sparse signals depends on prior knowledge of signal sparsity. When applying the OMP algorithm to reconstruct the intermediate signals formed by the noisy signals received by antenna arrays, a key challenge arises in enhancing the algorithm’s resistance to noise interference and determining the sparsity of intermediate signals to optimize the construction of the sensing matrix [24]. Thus, addressing the challenging task of setting prior knowledge to determine the sparsity of intermediate signals and eliminating noise interference to achieve accurate reconstruction of noisy signals using the OMP algorithm becomes crucial.

This paper proposes an improved algorithm, DTM_OMP_ICA, for the orthogonal matching pursuit (OMP) algorithm under a masking strategy [25], based on independent component analysis (ICA) [26,27]. This algorithm enhances the traditional OMP framework. It does so by employing OMP algorithms based on two distinct observation bases to derive two different sparse representations of the input signal. These representations are then used as dual inputs to the ICA algorithm, aimed at bolstering the robustness of the signal’s sparse representation. Furthermore, the algorithm integrates a mean mask strategy to accomplish denoising reconstruction of the input signal. The goal of this novel signal processing approach is to ensure the robustness and stability of the algorithm while accurately reconstructing the intermediate frequency signals constituted by noisy echo signals received by array antennas. The organization of the paper is as follows: Section 1 introduces the research motivation, significance, and content. Section 2 provides an overview of the process strategy of the orthogonal matching pursuit algorithm. In Section 3, the improved algorithm based on the masking strategy for the orthogonal matching pursuit algorithm (DTM_OMP_ICA) is proposed, and its theoretical feasibility is validated through mathematical formula derivation. Section 4 analyzes the frequency characteristics of signals reconstructed by the DTM_OMP_ICA algorithm under different noise environments, verifying the accuracy and stability of the algorithm. Additionally, the DTM_OMP_ICA algorithm is applied to denoise and reconstruct simulated signals received by continuous modulation wave radar array antennas in noisy environments to explore its performance in practical applications. Section 5 summarizes the proposed algorithm and provides prospects for future research.

## 2. The Principle of CS

CS exploits the sparsity of a signal in a certain transformed domain to achieve signal reconstruction with only a small number of compressed projection data. The core idea of its reconstruction algorithm is to project the signal into a lower-dimensional space and then solve an optimization problem to reconstruct the original signal. The objective of this optimization problem is to find the sparsest representation of the signal, minimizing the difference between the projected data and the original signal. In this way, compressed sensing can significantly reduce sampling and storage requirements while maintaining a high level of reconstruction quality.

### 2.1. The Sampling Process of Compressed Sensing

According to compressed sensing theory, signal compression encoding does not directly measure the signal itself. Instead, it involves projecting the signal onto a set of measurement matrices to obtain observed values. A one-dimensional signal, denoted as x (with a length of N), can be represented using an orthogonal transform basis (sparse basis) ψ and sparse representation θ, where the sparsity level is K, as shown in (1):

(1) $$x={\left(\psi \times \theta \right)}^{T}$$

where ψ∈N×N,θ∈N×1, and “${\left(\cdot \right)}^{T}$” represent the matrix transpose operation.

Assuming an observation basis φ, which is uncorrelated with the transform basis ψ, allows for the compression of the signal x. This compression is achieved by applying φ, resulting in a one-dimensional observation signal y of length M. Consequently, this process compresses signal x from its original length N to M, as outlined in Equation (2).

(2) y=φ×x

where φ∈M×N,x∈N×1,y∈M×1.

To ensure precise signal reconstruction, the design of the observation basis φ requires that during the transformation of the signal from x to y, the M observed values obtained should not destroy the essential information of the original signal x. This means that the number of observed values M should satisfy the following equation, and it should hold that K≪M≪N:

(3) M≥K×log⁡NK

Combining (1) and (2), we can obtain (4):

(4) y=φ×x=φ×ψ×θ=A×θ

where A represents the sensing matrix. A=φ×ψ,AϵM×N.

Due to the fact that the number of equations represented by (4) is much smaller than the number of unknowns, the solutions are non-unique, affecting the precise reconstruction of the signal x. However, because the signal x is k-sparse in its orthogonal transform basis ψ, if the φ in (4) satisfies the restricted isometry property (RIP) [28,29], then the signal x can be accurately reconstructed by the optimal solution from M observed values. The equivalent condition for RIP property is that the observation basis φ and the sparse basis ψ are uncorrelated.

### 2.2. Compressed Sensing Signal Reconstruction Algorithm

When the observation basis φ satisfies the RIP criterion, the compressed sensing signal reconstruction algorithm can obtain the sparse representation θ of the signal through solving the inverse problem of (4). Subsequently, it can precisely reconstruct the length-N signal x with K sparsity in the orthogonal transform basis ψ from the length-M observation vector y. Its mathematical expression is:

(5) $$\underset{\theta }{min}{\left\|\theta \right\|}_{0}\;\;s.t.\;\;y=A\times \theta$$

Currently, to effectively solve for θ to achieve signal reconstruction, algorithms are mainly divided into three major categories: convex optimization algorithms [30,31,32], greedy matching pursuit methods, and combination algorithms [33].

Among them, the greedy matching pursuit method iteratively searches for the support set of the sparse vector and reconstructs the signal using a constrained support least-squares estimate. Due to its low complexity, this algorithm can rapidly reconstruct signals and is suitable for real-time computing scenarios. Such algorithms mainly include matching pursuit (MP) [34], orthogonal matching pursuit (OMP), the compressive sampling matching pursuit (CoSaMP) algorithm [35], and the regularized OMP (ROMP) [36,37] algorithm, among others. However, these algorithms are sensitive to noise, and their noise resistance performance can be improved by combining them with other methods, such as preprocessing with wavelet transforms [38] or improving the sparse representation of signals through various dictionary learning methods [39].

### 2.3. Orthogonal Matching Pursuit (OMP)

The core challenge of OMP lies in determining which column $a_{i}$ of the matrix A contribute significantly to the vector y in (6). The distance between two vectors can be measured using the inner product of vectors. Therefore, vector y can be projected onto the column vectors of matrix A, and the numerical value of the inner product is used to assess the contribution of the column vectors of matrix A to vector y.

(6) $$Contribution\left(y,a_{i}\right)=\left|\frac{\left\langle y,a_{i}\right\rangle }{\left|a_{i}\right|}\right|$$

where $a_{i}$ represents the i-th column of matrix A. θ in (4), as the projection of signal x onto its orthogonal transformation basis ψ, is K-sparse; therefore, the algorithm only needs to iterate K times to find the K column vectors in matrix A that contribute the most to y, along with their positions. This allows for solving the current optimal solution θ to complete the reconstruction of signal x. The above describes the workflow of the orthogonal matching pursuit algorithm, and the pseudo-code for the algorithm is shown as follows:

In the pseudo-code shown in Algorithm 1, the input A represents the sensing matrix, y is a one-dimensional observation vector of length M, and K denotes the sparsity level of the original signal x (typically, the choice of K is related to the energy distribution of the one-dimensional signal x in its orthogonal transform basis ψ). The output θ represents the sparse representation of the signal x.
**Algorithm 1:** Orthogonal matching pursuit (OMP)**Input**: A,y,K**Output**: θ1 **Initialization**
r0=θ, $\Lambda_{0}=\emptyset ;$2 Normalize all columns of A to unit L_2_ norm;3 **for** k = 1,2, …, K **do**4            Step 1:      $\lambda_{k}{=}_{j\notin \Lambda_{k-1}}^{\arg\max\left|\left\langle a_{j},r_{\kappa -1}\right\rangle \right|};$5            Step 2:      $\Lambda_{k}=\Lambda_{K-1}\cup \lambda_{k};$6            Step 3:      $x_{k}\left(\mathrm{i}\in \Lambda_{k}\right){=}_{x}^{\arg\min{\left\|A_{\Lambda_{k}}x-y\right\|}_{2}};$7            Step 4:      $\tilde{\theta_{k}}=Ax_{k};$8            Step 5:      $\gamma_{k}=\theta -\tilde{\theta_{k}};$9 **end**

The above algorithm illustrates that the composition of the sensing matrix A is crucial for the reconstruction performance of the orthogonal matching pursuit algorithm [23]. It has been proven [40] that independently and identically distributed Gaussian random measurement matrices can serve as universal compressed sensing observation basis. Therefore, random Gaussian matrices are commonly used as observation basis. In addition to random Gaussian matrices, other commonly used observation basis include random Bernoulli matrices [41], random orthogonal matrices [42], Toeplitz matrices [43], and sparse random matrices [44], among others.

The theory of compressed sensing (CS) and its applications in denoising and signal reconstruction continue to garner interest among researchers. Particularly, due to its efficacy in processing sparse signals, the orthogonal matching pursuit (OMP) algorithm has emerged as a focal point of study. In environments characterized by the presence of noise, the robust recovery algorithm proposed by V Meena and G Abhilash, which improves upon the OMP algorithm, has demonstrated the effectiveness of compressed sensing under conditions of high signal-to-noise ratios [45]. Similarly, TJ Thomas and colleagues developed a new algorithm that not only enhances the recovery capabilities of OMP but also offers a new direction for the denoising of ECG signals [46].

Further, the comprehensive review by L Li et al. evaluates the potential applications of the OMP algorithm and its variants in noisy settings, providing valuable perspectives on the feasibility of compressed sensing across various application scenarios [47]. Moreover, the research by C Cheng and D Lin, applying the OMP algorithm in the process of image restoration, showcases the potential and challenges of compressed sensing theory in the field of image processing [48].

These developments indicate that while compressed sensing and the OMP algorithm have proven effective in multiple application scenarios, signal reconstruction in high-noise environments remains a challenge. To address these challenges effectively, the DTM_OMP_ICA algorithm introduced in this paper differs from traditional OMP algorithms in that it does not rely on a priori assumptions about signal sparsity. The algorithm can adaptively determine the sparsity level of the input signal, enhancing its flexibility and efficiency in processing signals with unknown or variable sparsity. Furthermore, by employing a threshold masking technique, the DTM_OMP_ICA algorithm significantly improves the accuracy and stability of signal reconstruction in noisy conditions.

## 3. Dual-Threshold Mask OMP Based on ICA

### 3.1. Principle of ICA

Independent component analysis (ICA) [24,25] is a multivariate statistical analysis method that aims to separate mutually independent components from mixed multidimensional data based on the independence assumption. Its mathematical expression is shown in (7).

(7) S=W×X

where S represents the data matrix of independent components, X is the original observation matrix, and W is the mixing matrix used to transform the original data X into the independent components S.

In the ICA algorithm model, the core objective is to estimate the mixing matrix W. This objective is achieved by maximizing the independence between components. Typically, the independence between components is measured through information entropy or higher-order statistics, serving as a guide for extracting independent components. In practical scenarios, noisy signals are often a mixture of source signals and additive noise. In other words, the original observation matrix X in (7) can be represented as follows:

(8) $$X=\alpha_{1}x_{1}+\alpha_{2}x_{2}$$

where X represents the noisy signal, $x_{1}$ is the pure signal, $x_{2}$ is the additive noise, $\alpha_{1}$ and $\alpha_{2}$ represent the energy proportions of the pure signal and additive noise in the noisy signal X, and their values are determined by the signal-to-noise ratio of the noisy signal X.

Assuming that the clean signal $x_{1}$ and additive noise $x_{2}$ come from mutually independent, different sources, which satisfy the independence assumption of the ICA algorithm, this allows for the ICA algorithm to effectively capture the independence information between the original signal and additive noise in the noisy signal by estimating the mixing matrix W in (8). This provides strong support for signal denoising processing.

### 3.2. DTM_OMP_ICA

When using the OMP algorithm to reconstruct the noisy signal X in (8), its observed that signal y can be expressed as follows:

(9) $$y=\varphi \times X=\varphi \times \left(\alpha_{1}x_{1}+\alpha_{2}x_{2}\right)=A\times \theta$$

where φ, $\alpha_{1}$, $\alpha_{2}$, A, θ, $x_{1}$, and $x_{2}$ have the same meanings as defined in (2), (4), and (8).

In the theory of compressed sensing, random matrices are commonly chosen as the observation basis φ, and the Fourier transform matrix is used as the orthogonal transform basis ψ. Moreover, for the observation basis φ, the number of rows M and the number of columns N satisfy (3). This implies that the row vectors of the observation basis φ are usually linearly independent, making the observation basis φ typically full-rank in rows. Additionally, the Fourier transform basis, being the Fourier transform of the identity matrix E, is also a full-rank matrix. Therefore,

(10) $$r\left(\varphi \right)=M,\;\;\;\;\;\;r\left(\psi \right)=N$$

At the same time, the sensing matrix A=φ×ψ. Therefore, it is inevitable that the sensing matrix A is a full-rank matrix in rows, i.e.,

(11) $$r\left(A\right)=r\left(\varphi \right)=M$$

For sensing matrix A, there must exist a pseudo-inverse matrix $A^{+}$ such that (12) and (13) hold as follows:

(12) $$A\times A^{+}\times A=A$$

(13) $$A^{+}\times A\times A^{+}=A^{+}$$

Therefore, (9) can be rewritten as

(14) $$\theta =A^{+}\times y=A^{+}\times \varphi \times \left(\alpha_{1}x_{1}+\alpha_{2}x_{2}\right)=C\times \left(\alpha_{1}x_{1}+\alpha_{2}x_{2}\right)$$

where $C=A^{+}\times \varphi$.

Since the term C in (14) involves only matrix correlations, the sparse representations ($\theta_{RGM}$, $\theta_{SRM}$) obtained by OMP algorithms based on two different random matrices as observation bases ($\varphi_{RGM}$, $\varphi_{SRM}$) for the noisy signal X can be considered as different linear transformations of the noisy signal X, as shown in (15) and (16):

(15) $$\theta_{RGM}=A_{RGM}^{+}\times \varphi_{RGM}\times \left(\alpha_{1}x_{1}+\alpha_{2}x_{2}\right)=C_{RGM}\times \left(\alpha_{1}x_{1}+\alpha_{2}x_{2}\right)$$

(16) $$\theta_{SRM}=A_{SRM}^{+}\times \varphi_{SRM}\times \left(\alpha_{1}x_{1}+\alpha_{2}x_{2}\right)=C_{SRM}\times \left(\alpha_{1}x_{1}+\alpha_{2}x_{2}\right)$$

These distinct sparse representations of the noisy signal X correspond to different weighted mixtures of the clean signal $x_{1}$ and the additive noise $x_{2}$. Simultaneously, $x_{1}$ and $x_{2}$ originate from independent sources, satisfying the independence assumption of the ICA algorithm. Therefore, $\theta_{RGM}$ and $\theta_{SRM}$ can be used as inputs to the ICA algorithm. Leveraging the ICA algorithm allows for obtaining sparse representations, namely, $\theta_{1}$ related solely to the clean signal $x_{1}$ and $\theta_{2}$ related exclusively to the additive noise $x_{2}$, from the noisy signal X. Subsequently, $\theta_{1}$ will be utilized for denoising and signal reconstruction of the noisy signal X.

**Figure 1:**
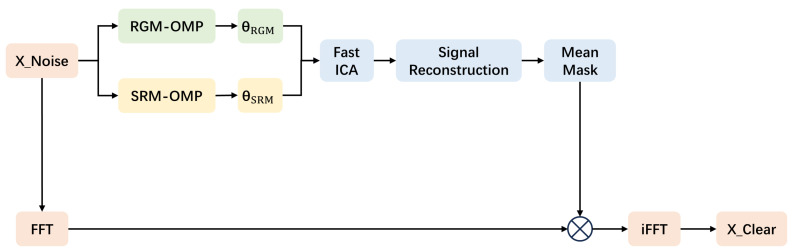
DTM_OMP_ICA.

Based on the above theory, the present paper proposes the DTM_OMP_ICA algorithm to eliminate or reduce the impact of noise on signal reconstruction. The flowchart of the DTM_OMP_ICA algorithm is shown in Figure 1.

In Figure 1, when denoising and reconstructing the noisy signal X_Noise, the proposed DTM_OMP_ICA algorithm employs OMP algorithms based on two different observation bases (RGM-OMP and SRM-OMP) to construct a dual-path process. This results in obtaining distinct sparse representations, namely, $\theta_{RGM}$ and $\theta_{SRM}$, for the noisy signal X_Noise. The algorithm combines the usage of the ICA algorithm (Fast ICA) to decouple and obtain sparse representations most correlated with the X_Clear component in the noisy signal X_Noise, achieving denoising and reconstruction of X_Noise. To ensure that the reconstructed signal retains the frequencies and corresponding amplitudes of X_Clear from X_Noise as much as possible, a mean mask technique is applied based on the frequency domain mean of the reconstructed signal. This technique generates a mask matrix suitable for the frequency domain of X_Noise, multiplies it with the spectrum of X_Noise, and employs inverse Fourier transform for denoising and reconstructing the noisy signal X_Noise.

The pseudo-code for DTM_OMP_ICA is shown as follows:

In the pseudo-code shown in Algorithm 2, “path 1” and “path 2” represent the dual-path process constructed using OMP algorithms with different observation bases. “Orth(rand(·))” is utilized to generate a random Gaussian matrix. “SparseRandomMtx(·)” is employed to generate a sparse random matrix. “OMP(·)” indicates the use of the OMP algorithm.
**Algorithm 2:** Dual-threshold mask OMP based on ICA (DTM_OMP_ICA)**Input:** X_Noise**Output:** Reconstructed X_Clear1 **Initialization**
K=0, M=0, N=len(X_Noise);2 **do**3      Step 1:  K=OutLine_Extra(X_Noise);4      Step 2:  M=K×log⁡(N/K);5      Step 3:  $\psi =\frac{1}{\sqrt{N}}\times FFT(I)$;6      **path1 do**7          Step 1:  $\varphi_{RGM}=Orth(rand(M,N))$;8          Step 2:  $y_{RGM}=\varphi_{RGM}\times X\_Noise$; $A_{RGM}=\varphi_{RGM}\times \psi$;9          Step 3:  $\theta_{RGM}=OMP(A_{RGM},y_{RGM},K)$;10     **end path1**11     **path2 do**12          Step 1:  $\varphi_{SRM}=SparseRandomMtx(M,N,d=4)$;13          Step 2:  $y_{SRM}=\varphi_{SRM}\times X\_Noise$; $A_{SRM}=\varphi_{SRM}\times \psi$;14          Step 3:  $\theta_{SRM}=OMP(A_{SRM},y_{SRM},K)$;15    **end path2**16      Step 4:  $\theta_{1}=corr(FastICA(\theta_{RMG},\theta_{SRM}))$;17      Step 5:  $Reconstructed\_X=\psi^{T}\times {\theta_{1}}^{T}$;18      Step 6:  Mask=MeanMask(FFT(Reconstructed_X));19      Step 7:  FX=dot(FFT(X_Noise), Mask);20      Step 8:  Reconstructed X_Clear=iFFT(FX);21 **end**

The pseudo-code function OutLine_Extra represents the outlier detection algorithm [49], used to identify anomalies in the signal spectrum and determine the signal’s sparsity level. Its definition is as follows:

(17) $$OutLine\_Extra≝\frac{1}{N}\sum_{i=1}^{N}\left|X_{i}-median\left(X\right)\right|$$

where N represents the size of the data to be examined X, $X_{i}$ denotes the i-th sample point in the data X.

The function MeanMask represents the generation of a mask matrix using the frequency domain mean of the input signal. It is defined as follows:

(18) $$MeanMask\left(i\right)≝\left\{\begin{array}{c} 0,\;\;\;\;\;\;Fx\left(i\right)-mean(Fx)<0\\ 1,\;\;\;\;\;\;Fx\left(i\right)-mean(Fx)\geq 0 \end{array}\right.$$

where “Mask” represents the generated mask matrix; “Fx” is used to denote the spectrum of the noisy signal x after Fourier transformation, and “Fx” and “Mask” have the same length; “mean(·)” indicates the mean calculation; and “i” represents the i-th spectral line.

## 4. Experiments and Results

This paper conducted two sets of simulation experiments. The first set evaluated the reconstruction performance of a noisy multi-frequency signal, focusing on accuracy, stability, and processing time. The second set involved the reconstruction experiment of a noisy array antenna. The comparative algorithms for both sets were the RGM-OMP and SRM-OMP algorithms. Each experiment category consisted of 100 rounds of independent repetitions.

### 4.1. Multi-Frequency Signals with Noise Experiment

#### 4.1.1. Data

The experiment simulated a multi-frequency signal with four different frequencies and added noise with varying SNR. This was carried out to validate the stability and accuracy of the proposed DTM_OMP_ICA algorithm in denoising and reconstructing multi-frequency signals in the presence of noise.

The multi-frequency signal used in this paper was represented as:

(19) $$x^{\prime }=\cos\left(2\pi f_{1}t\right)+0.7\cos\left(2\pi 2f_{2}t\right)+1.6\cos\left(2\pi f_{3}t\right)+1.2\cos\left(2\pi f_{4}t\right)$$

where $f_{1}=500\;Hz,\;f_{2}=1000\;Hz,\;f_{3}=1500\;Hz,\;f_{4}=2000\;Hz$.

The main parameter settings of the algorithm were shown in Table 1.

**Figure 2:**
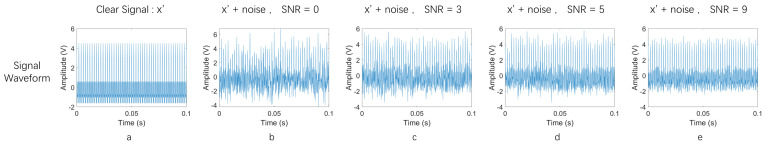
Time−domain waveform plots of the multi−frequency signal. (**a**) Original clean multi−frequency signal $x^{\prime }$. (**b**) Noisy multi−frequency signal x at 0 dB SNR. (**c**) Noisy multi−frequency signal x at 3 dB SNR. (**d**) Noisy multi−frequency signal x at 5 dB SNR. (**e**) Noisy multi−frequency signal x at 9 dB SNR.

The noisy signal used in this paper is represented as

(20) $$x=x^{\prime }+noise$$

where noise represents Gaussian white noise. Four sets of noisy signals with different SNRs (0 dB, 3 dB, 5 dB, and 9 dB) were used to compare in the experiments. The time-domain waveforms of all multi-frequency signals used in this paper are shown in Figure 2.

In Figure 2, the time-domain waveform graphs from left to right represent the clean multi-frequency signal $x^{\prime }$ and the noisy multi-frequency signal x at SNRs of 0 dB, 3 dB, 5 dB, and 9 dB, respectively.

#### 4.1.2. Results of Multi-Frequency Signal with Noise

##### Spectral Analysis of Reconstructed Signals

**Figure 3:**
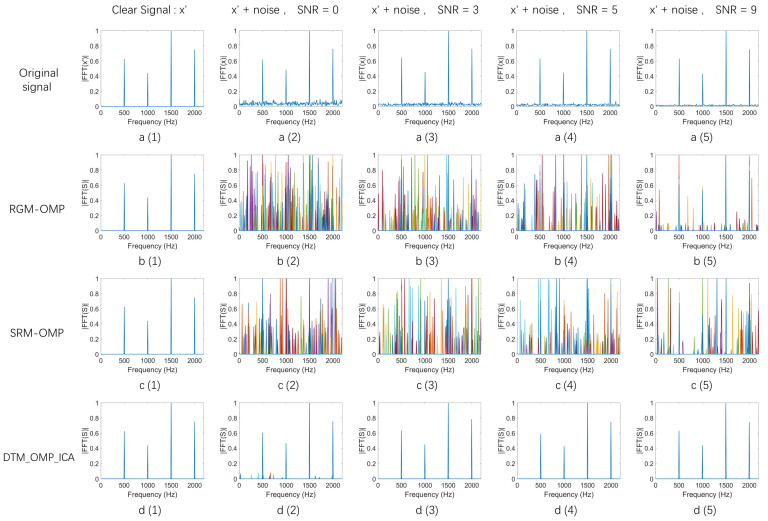
The spectrum of signals reconstructed by different algorithms. (**a(1)**–**a(5)**) Spectrum of the original multi-frequency signal $x^{\prime }$ and its noisy counterparts at SNRs of 0 dB, 3 dB, 5 dB, and 9 dB, respectively. (**b(1)**–**b(5)**) Spectrum of the signal reconstructed by RGM-OMP under the same SNRs. (**c(1)**–**c(5)**) Spectrum of the signal reconstructed by SRM-OMP under the same SNRs. (**d(1)**–**d(5)**) Spectrum of the signal reconstructed by DTM_OMP_ICA under the same SNRs.

After 100 rounds of independent repeated experiments, the spectra of the reconstructed signals S using three different algorithms for the signals at the mentioned five different SNRs were shown in Figure 3.

Figure 3 presents a comparative analysis of the signal reconstruction capabilities of RGM-OMP, SRM-OMP, and the DTM_OMP_ICA algorithm proposed in this paper under various noise conditions. The first row of five spectral graphs (Figure 3(a(1)–a(5))) displays the spectrum of the multi-frequency signal $x^{\prime }$ and its noisy counterparts x under different signal-to-noise ratios (SNRs of 0 dB, 3 dB, 5 dB, and 9 dB, respectively). Following these, the next three rows correspond to the spectra of the reconstructed signal S after processing by the RGM-OMP, SRM-OMP, and DTM_OMP_ICA algorithms, respectively, for the aforementioned multi-frequency signals.

Figure 3 clearly demonstrates that in an ideal noise-free environment, all three algorithms exhibit excellent reconstruction performance, successfully retaining the original frequency components of the signal in the spectrum of the reconstructed signal S (as shown in Figure 3(b(1),c(1),d(1))). However, as the noise level increases, the reconstructed signal spectra of the RGM-OMP and SRM-OMP algorithms begin to show evident frequency components arising from noise interference (as depicted in Figure 3(b(2)–b(5),c(2)–c(5))), and the number of interference frequency components appearing in the spectra of the signals reconstructed by the RGM-OMP and SRM-OMP algorithms increases with the noise, largely due to the randomness of the observation basis they employ. In contrast, the signal reconstructed by the DTM_OMP_ICA algorithm not only retains the integrity of the original pure signal $x^{\prime }$ frequency components in its spectrum from the noisy multi-frequency signal x but also significantly reduces the frequency components arising from noise interference (as shown in Figure 3(d(2)–d(5))). The experimental results indicate that the DTM_OMP_ICA algorithm surpasses traditional OMP algorithms in robustness when reconstructing noisy multi-frequency signals.

##### Evaluations of Reconstructed Signals

To objectively assess the noise-resistant reconstruction stability of the algorithm, this paper proposed the following stability evaluation.

(i) Evaluation of Signal Reconstruction Accuracy
  This paper employs statistical parameters ((21)–(23)) to perform algorithm stability analysis.
  A. The accuracy of reconstructed signal frequencies, denoted as AccF:
  (21) $$AccF=\left(\frac{AccFNum}{TotalNum}\right)\times 100\%$$
  where AccFNum represents the number of times the correct frequencies appear in the reconstructed signal and TotalNum is the total number of frequency components in the reconstructed signal.
  B. The error rate of reconstructed signal frequencies, denoted as ErrF:
  (22) $$ErrF=\left(\frac{ErrFNum}{TotalNum}\right)\times 100\%$$
  where ErrFNum represents the number of times incorrect frequencies appear in the reconstructed signal.
  C. The accuracy of both the frequency and amplitude of the reconstructed signal, denoted as AccFA:

(23) $$AccFA=\left(\frac{AccFANum}{AccFNum}\right)\times 100\%$$

where AccFANum represents the number of times both the correct frequency and amplitude appear in the reconstructed signal.

**Figure 4:**
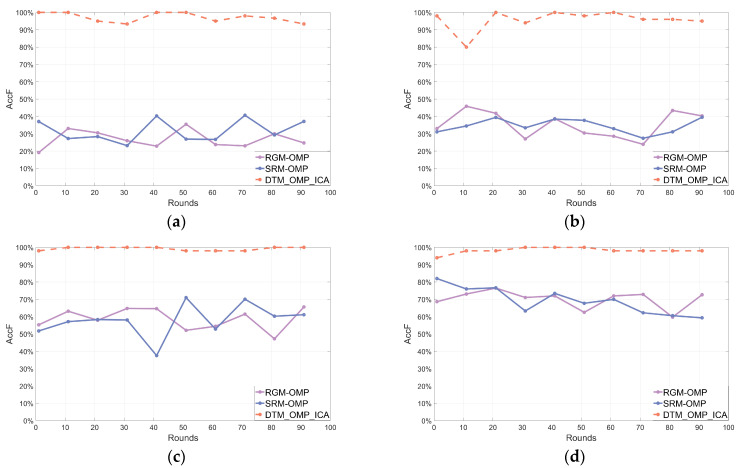
Reconstructed frequency statistical parameter AccF. (**a**) SNR = 0. (**b**) SNR = 3. (**c**) SNR = 5. (**d**) SNR = 9.

**Figure 5:**
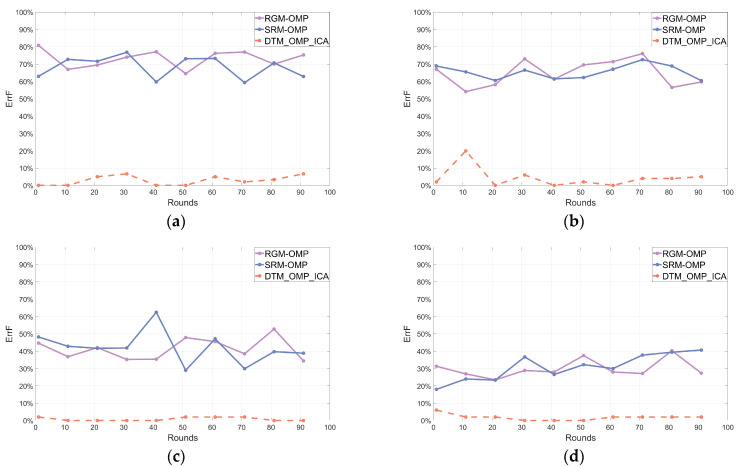
Reconstructed frequency statistical parameter ErrF. (**a**) SNR = 0. (**b**) SNR = 3. (**c**) SNR = 5. (**d**) SNR = 9.

**Figure 6:**
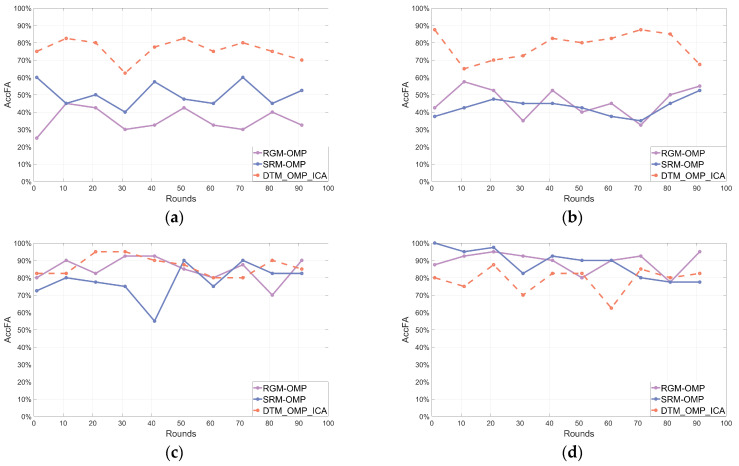
Reconstructed frequency statistical parameter AccFA. (**a**) SNR = 0. (**b**) SNR = 3. (**c**) SNR = 5. (**d**) SNR = 9.

After 100 rounds of independent repeated experiments, the line charts illustrating the frequency statistical parameters of the reconstructed signals using three different algorithms are shown in Figure 4, Figure 5 and Figure 6, respectively.

Figure 4, Figure 5 and Figure 6 depicted line charts of frequency statistical parameters, including AccF, ErrF, and AccFA, for the signals reconstructed by three different algorithms in 100 rounds of independent repeated experiments. Subplots (a–d) in Figure 4, Figure 5 and Figure 6 corresponded to the frequency statistical parameters of the reconstructed signals for different SNRs of the noisy multi-frequency signals x (SNR of 0 dB, 3 dB, 5 dB, and 9 dB, respectively) using each algorithm. In each subplot, the orange dashed line represents the proposed DTM_OMP_ICA algorithm, while the purple and blue solid lines correspond to the RGM-OMP and SRM-OMP algorithms, respectively. To simplify and visualize the frequency statistical parameters of the reconstructed signals, the values of the parameters shown in (18), (19), and (20) were averaged every 10 rounds of independent repeated experiments.

Figure 4, Figure 5 and Figure 6 indicated that the reconstruction performance of the traditional OMP algorithm was affected by changes in the SNR values of the noisy multi-frequency signals. In comparison, the algorithm proposed in this paper exhibited a relatively stable denoising and reconstruction capability for noisy multi-frequency signals, and it had a higher probability of reconstructing the frequency components of the pure signals within the noisy multi-frequency signals.

**Figure 7:**
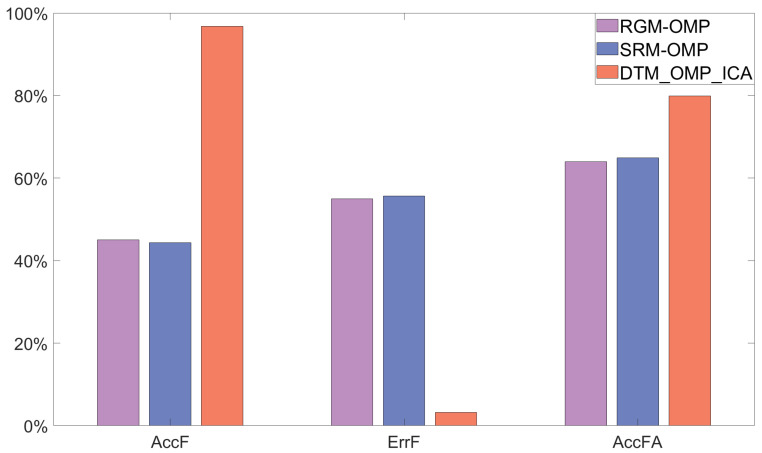
Overall analysis of reconstructed frequency statistical parameters for three algorithms.

After 100 independent repeated experiments, this paper conducted an overall analysis of the frequency statistical parameters of the reconstructed signals using three different algorithms at different SNRs, as shown in Figure 7.

In Figure 7, the *x*-axis represents three different frequency statistical parameters, and the *y*-axis represents the mean values of frequency statistical parameters of signals reconstructed by different algorithms after 100 rounds of independent repeated experiments with four different signal-to-noise ratios (SNRs) for noisy multi-frequency signals. The purple, blue, and orange bars represent the RGM-OMP, SRM-OMP, and DTM_OMP_ICA algorithms, respectively.

The results shown in Figure 7 indicate that the proposed DTM_OMP_ICA algorithm excelled in the accuracy of reconstructing signal frequencies (AccF), achieving an average accuracy of 96.8%, significantly surpassing RGM-OMP with 45.01% and SRM-OMP with 44.38%. In terms of the error rate of reconstructed signal frequencies (ErrF), DTM_OMP_ICA was at 3.18%, much lower than RGM-OMP at 54.99% and SRM-OMP at 55.62%. Additionally, DTM_OMP_ICA attained an accuracy of 79.88% in reconstructing the frequencies and amplitudes of signals (AccFA), surpassing other algorithms by 15.94% and 15%, respectively.

(ii) Evaluation of Signal Reconstruction Stability

**Figure 8:**
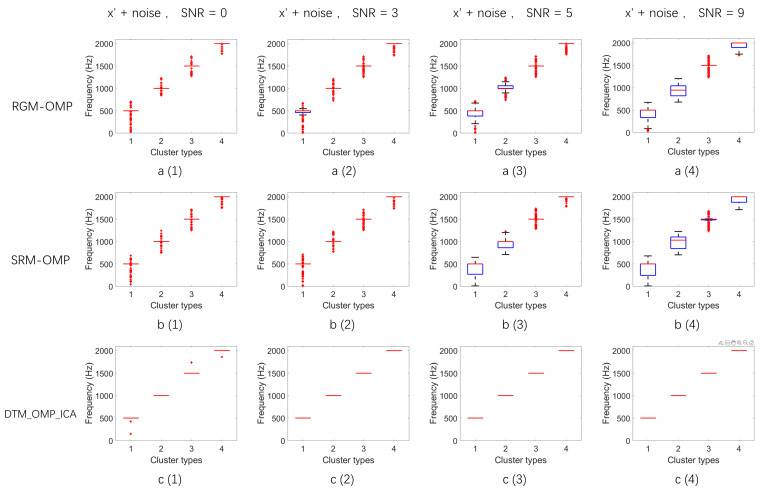
Frequency clustering statistical results. (**a(1)**–**a(4)**) Frequency clustering results of the reconstructed signal S using RGM-OMP at SNRs of 0 dB, 3 dB, 5 dB, and 9 dB, respectively. (**b(1)**–**b(4)**) Frequency clustering results of the reconstructed signal S using SRM-OMP at the same SNRs. (**c(1)**–**c(4)**) Frequency clustering results of the reconstructed signal S using DTM_OMP_ICA at the corresponding SNRs.

The mean and variance of the reconstructed frequencies implemented by the algorithm were statistically analyzed using 100 experiments. First, k-means clustering was used to cluster the reconstructed frequencies obtained by the algorithm. Since the signal X was a linear combination of four frequencies, the number of clusters was set to 4. The statistical results of the three algorithms mentioned above are shown in Figure 8.

Figure 8 meticulously illustrates the frequency clustering results of the reconstructed signal S after processing the noisy multi-frequency signal x through RGM-OMP, SRM-OMP, and the DTM_OMP_ICA algorithm proposed in this paper under different signal-to-noise ratio (SNR) environments (0 dB, 3 dB, 5 dB, and 9 dB). Each row of four subfigures corresponds to the same algorithm, with the three rows of subfigures from top to bottom, respectively, showcasing the frequency clustering results of the reconstructed signal S after using the RGM-OMP, SRM-OMP, and DTM_OMP_ICA algorithms (for example: Figure 8(c(1)) displays the frequency clustering result of the reconstructed signal S after processing through the DTM_OMP_ICA algorithm in a noise environment with an SNR of 0 dB).

A comparative analysis of the results shown in each subfigure of Figure 8 clearly reveals that after reconstructing the noisy multi-frequency signal x using traditional OMP signal reconstruction algorithms based on two different observation bases, the frequency clustering results of the reconstructed signal S demonstrate that the average value of each frequency cluster deviates from the frequency of the original noise-free signal. Additionally, the variance within each frequency cluster in the clustering results is considerable, especially more pronounced in environments with stronger noise interference. For example, in a noise environment with an SNR of 0 dB, the frequency distribution variance within the four frequency clusters of the signal reconstructed by the RGM-OMP algorithm are 32,355, 24,389, 10,043, and 7251, as shown in Figure 8(a(1)), where each frequency cluster is surrounded by numerous outlier frequencies with a wide distribution range. Similarly, in the frequency clustering results of the signal reconstructed by the SRM-OMP algorithm shown in Figure 8(b(1)), each frequency cluster is also surrounded by outlier frequencies with a large distribution range, with variances within each cluster of 10,587, 12,967, 19,081, and 4697. These experimental results indicate that the stability of the frequency distribution of the reconstructed signal weakens as noise interference increases after reconstructing the noisy multi-frequency signal using traditional OMP algorithms.

In contrast, the frequency clustering results of the signal S reconstructed by the DTM_OMP_ICA algorithm proposed in this paper show that the average value of each frequency cluster is closer to the frequency of the original noise-free signal, and the variance within the four frequency clusters is near zero. Even in a strong noise interference environment with an SNR of 0 dB, the frequency clustering results of the signal reconstructed by the DTM_OMP_ICA algorithm demonstrate that the average values of the four frequency clusters are closer to the frequencies of the original noise-free signal, with the variance within each cluster being significantly low (0.07, 0, 0.0042, 0.005), as illustrated in Figure 8(c(1)). The occurrence of outlier frequencies within each clustered frequency is greatly reduced compared to Figure 8(a(1),b(1)). Similarly, under noise interference of 3 dB, 5 dB, and 9 dB, the frequency clustering results of the signal reconstructed by the DTM_OMP_ICA algorithm show that the average value within each frequency cluster matches the original noise-free signal frequency exactly, and the variance is closer to zero. The experimental results indicate that the DTM_OMP_ICA algorithm’s stability in reconstructing noisy multi-frequency signals surpasses that of traditional OMP algorithms.

Based on the observations, it can be concluded that the DTW_OMP_ICA algorithm proposed in this paper exhibited superior noise resistance and stability compared to traditional OMP algorithms. In noisy environments, it excelled at reconstructing the original signal frequencies more accurately.

(iii) Time Consumption

In order to exclude the effect of computer hardware chance on the running time of the algorithm, the experiment was conducted with 10 sets of 100 rounds of independent repetitive experiments with the same settings as before. The computer was configured with CPU: AMD Ryzen 7 5800H (8 cores, 16 threads, 3.2 GHz); RAM: DDR4-3200 16 GB; and the final algorithmic runtime results were averaged over the 10 groups of recorded durations, and counted using the durations of a single round, ten rounds, fifty rounds, and one hundred rounds of different algorithmic-based runs, the results of which are shown in Table 2.

Table 2 shows the statistics of the length of time the algorithms were run independently, where the first column shows the three different algorithms used, and columns 2, 3, 4, and 5 show the time consumed by each algorithm in different rounds of independently repeated experiments.

Table 2 indicates that the RGM-OMP algorithm had the lowest time cost for reconstructing noisy multi-frequency signals, followed by the SRM-OMP algorithm, but the difference is minimal. The proposed algorithm, which employs both RGM-OMP and SRM-OMP to construct a dual-path process and combines them with ICA to find the most relevant sparse representation of the clean signal in the noisy signal, incurred the highest time cost. However, it was still less than the sum of the time taken by the RGM-OMP and SRM-OMP algorithms.

While the DTM_OMP_ICA algorithm incurred a time cost approximately 1.7 times that of the traditional OMP algorithm, considering its overall performance in denoising and reconstructing noisy multi-frequency signals, the DTM_OMP_ICA algorithm still maintained a certain advantage over the traditional OMP algorithm.

### 4.2. Array Antenna Signal Denoising Experiment

To verify the denoising and reconstruction performance of the proposed DTM_OMP_ICA in practical applications, this paper applies it to the denoising process of signals received by an array antenna. Furthermore, to comprehensively evaluate the performance of our algorithm in real-world scenarios, this paper also conducts a comparative analysis using two orthogonal matching pursuit algorithms based on different observation bases—RGM-OMP and SRM-OMP—as well as two classic signal denoising techniques—the singular value decomposition denoising algorithm [50] and the wavelet threshold denoising algorithm [51].

#### 4.2.1. Data

The array antenna signal data used in this paper were derived from the simulated data provided in Problem A of the 2022 China Postgraduate Mathematical Modeling Competition. The data were generated from the echo signals received by a simulated FMCW (frequency-modulated continuous-wave) radar with 86 array antennas in a noisy environment. The specific parameters of the signals received by the array antenna are shown in Table 3.

#### 4.2.2. Results of Simulated Radar Signal Processing

**Figure 9:**
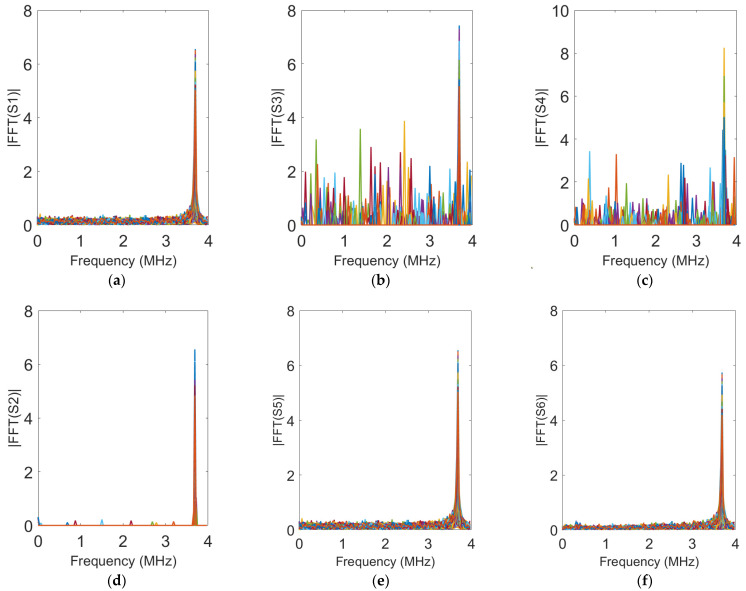
Spectrum of signals before and after reconstruction. (**a**) Original signal. (**b**) RGM-OMP. (**c**) SRM-OMP. (**d**) DTM_OMP_ICA. (**e**) SVD denoising. (**f**) Wavelet thresholding denoising.

This paper employed the proposed DTM_OMP_ICA algorithm, along with the RGM-OMP algorithm, SRM-OMP algorithm, SVD decomposition denoising algorithm, and wavelet threshold denoising algorithm used for comparative experiments, and applied them to denoise and reconstruct the noisy echo signals received by 86 array antennas in a simulated FMCW radar system. The reconstructed signals were then subjected to Fourier transform for frequency domain analysis, and the results are presented in Figure 9.

Figure 9a displays the spectral graph of the noisy echo signal S1 received by 86 array antennas. Subsequently, Figure 9b–f each present the spectral graphs of signals processed through various denoising algorithms, including RMG-OMP, SRM-OMP, DTM_OMP_ICA, SVD decomposition denoising, and wavelet threshold denoising. The signals reconstructed by these algorithms are, respectively, denoted as S3, S4, S2, S5, and S6.

Figure 9 indicates that, after denoising and reconstructing the noisy signals received by the array antennas using the DTM_OMP_ICA algorithm proposed in this paper, the reconstructed signals effectively reduced the majority of interference from noise while preserving the original frequency characteristics of the signals. Since the positioning performance of FMCW radar systems relies on the effective frequencies of received signals, the DTM_OMP_ICA algorithm could provide assistance in achieving precise localization of target objects for radar systems in noise-disturbed environments.

## 5. Conclusions and Discussion

This paper proposed the DTM_OMP_ICA algorithm to validate and eliminate the potential negative impact resulting from the strong randomness of the observation matrix used in the OMP algorithm. Unlike traditional OMP algorithms, the DTM_OMP_ICA algorithm does not directly reconstruct the signal. Instead, it generates a denoising mask matrix to perform denoising on the noisy signal. This approach effectively addresses the limitations of traditional OMP algorithms in noise resistance. The reconstructed signal, while preserving the original signal’s characteristics, reduces interference from most of the noise, making this algorithm significantly superior when reconstructing noisy signals.

This paper conducted comparative experiments by designing the same simulation experiments and contrasting them with the OMP reconstruction algorithm based on two different observation matrices. The experimental results demonstrate that the proposed method exhibits outstanding noise resistance, with a maximum noise resistance performance of up to 98%. Additionally, this algorithm showcases excellent stability, with a maximum stability performance of up to 99%, which is significantly better than traditional OMP algorithms. Admittedly, the above superior performance comes at the cost of time: the time consumption of the DTM_OMP_ICA algorithm is about 1.7 times that of two traditional algorithms. In practical applications, this method provides an innovative approach to the problem of reconstructing noisy signals and holds promising prospects in the relevant fields.

Additionally, the experiments found that the threshold setting for the mean mask in the proposed algorithm has an impact on the experimental results. When the threshold is set from 5 times to 30 times the mean energy of the reconstructed signal’s frequencies for constructing the mask matrix, the algorithm demonstrates good reconstruction performance. However, setting a threshold exceeding 50 times the mean energy of the reconstructed signal’s frequencies may result in the loss of some frequency features of the signal itself. On the other hand, setting a threshold less than five times the mean energy of the reconstructed signal’s frequencies for the mask matrix may lead to reduced noise resistance when reconstructing noisy signals with a high SNR. Therefore, effectively setting the mask threshold will be studied in our future work.

## Figures and Tables

**Table 1:** Initial parameter settings for the experiment.

Parameter	Value	Introduction
K	8	The sparsity of the signal in its orthogonal transformation basis (since the Fourier transform is two-sided, the sparsity is 4 × 2).
N	1024	The original number of sampled points in the signal.
t	128 (unit: ms)	Duration of the Signal.
fs	8000 (unit: Hz)	The signal’s sampling frequency based on the Nyquist sampling theorem will be used for subsequent frequency domain analysis of the reconstructed signal.

**Table 2:** Algorithm runtime summary.

	Round	Round 1	Round 10	Round 50	Round 100
Algorithm					
RGM-OMP		45.41 (ms)	227.62 (ms)	880.43 (ms)	1839.89 (ms)
SRM-OMP		46.43 (ms)	277.01 (ms)	926.09 (ms)	1915.21 (ms)
DTM_OMP_ICA		91.14 (ms)	414.41 (ms)	1446.20 (ms)	2785.59 (ms)

**Table 3:** Parameters and significance of signals received by array antennas.

Variables	Significance
$T=3.2\times 10^{-5}\;(s)$	Represents the chirp period, pulse time
$T_{s}=1.25\times 10^{-7}\;(s)$	Sampling interval
$N_{f}=32$	$N_{f}$ represents the number of chirp cycles
L=0.0815 (m)	The aperture of the array antennas
$\gamma =78.986\times 10^{12}\;(Hz/s)$	γ represents the frequency modulation slope
$N_{a}=86$	The number of equivalent virtual antenna arrays
$C=3\times 10^{8}\;(m/s)$	Speed of light
$f_{0}=78.8\times 10^{9}\;(Hz)$	Represents the carrier frequency

## Data Availability

The data used in this paper were sourced from the simulated intermediate-frequency (IF) noisy signals of the 2022 China Graduate Mathematical Modeling Competition, Problem A.
